# 

**Published:** 1845-05

**Authors:** 


### CONTENTS

OF NO. V.

### ORIGINAL COMMUNICATIONS.

### ESSAYS AND CASES.

ART. I.—Lectures on Physical Diagnosis. By J. R. BUCK, M. D., Lecturer on the Theory and Practice of Medicine in the Louisville Summer School of Medicine. - - - - 369

ART. II.—On the Use of Mercury in the Treatment of Southern Fevers. By LAFAYETTE CARR, M.D., of Aberdeen, Mississippi. - - - - - - - - - - - - - 379

ART. III.—A Case of Diseased Kidney, with the Post-mortem appearances, abridged from the Case-book of the Hospital of the Kentucky Penitentiary. By W. C. SNEED, M.D., of Frankfort, Ky. - - - - - - - - - - - - 383

ART. IV.—A Case of Empyema successfully treated by an Operation. By W. C. SNEED, M. D., of Frankfort, Kentucky. - - - - - - - - - - - - - 386

### REVIEWS.

ART. V.—An Essay on the Philosophy of Medical Science. By ELISHA BARTLETT, M.D., Professor of the Theory and Practice of Medicine in the University of Maryland. Philadelphia: Lea & Blanchard. 1844. 8vo. pp. 312. - 400

ART. VI.—A Treatise on Poisons in relation to Medical Jurisprudence, Physiology, and the Practice of Physic. By ROBERT CHRISTISON, M.D., F.R.S.E.; Professor of Materia Medica in the University of Edinburgh; Fellow of the Royal College of Physicians, &c.; Member of the American Philosophical Society, of the Royal Academy of Medicine of Paris, &c., &c., &c. First American, from the Fourth Edinburgh Edition. Philadelphia: Ed. Barrington & Geo. D. Haswell. 1845. 8vo. pp. 756. - 413

SELECTIONS FROM AMERICAN AND FOREIGN JOURNALS

Rupture of the Uterus and Abdominal Parietes. Cæsarean Operation performed with Success, both for the Mother and Infant. Thirteen months afterwards, Rupture of Uterus and Abdominal Parietes, from a second pregnancy: Birth of the Infant by this spontaneous opening, and perfect Recovery of the Mother. By Dr. PEARL, Director of the Obstetrical Institution of the city of Hildesheim. - - 429
Color of the Blood, - - - - - - - - - 433
Animal Heat, - - - - - - - - - - - 436
Analysis and Properties of the Gastric Fluid, - - - - 439
Digestive Properties of the Gastric Fluid, - - - - 439
The Physical Position of Man in Creation, - - - - 441
Raciborski on Puberty, - - - - - - . - - 442
Festival to commemorate the Introduction of the Potato, - - 444
Proportion of the Insane, - - - - - - . - - 445
Dr. Home, - - - - - - - - - - 445
Hooping-Cough, its Treatment and Effects, - - - - 446
Belladonna in Hooping-Cough, - - - - - - - - 446
Prognosis of Chancre, with reference to the Probabilities of Secondary Symptoms, - - - - - - - - - 448
Exemption of the Cherokee Indians and Africans from Insanity, 449
The Engrafting of Nerves. - - - - - - - - 449
On the Incubation of Insanity, - - - - - - - 450
Jewish Physicians in Hamburgh, - - - - - - 451
Enlargement of the Breasts in a Male: Atrophy of the Genital Organs, - - - - - - - - - - - 452

ORIGINAL INTELLIGENCE.

Longevity of Medical Men, - - - - - - - - 453
Asafœtida a Preventive of Measles, - - - - - - 455
A Question of Legitimacy, - - - - - - - 457
Books Received, - - - - - - - - - - 460

